# K65R in Subtype C HIV-1 Isolates from Patients Failing on a First-Line Regimen Including d4T or AZT: Comparison of Sanger and UDP Sequencing Data

**DOI:** 10.1371/journal.pone.0036549

**Published:** 2012-05-16

**Authors:** Patricia Recordon-Pinson, Jennifer Papuchon, Sandrine Reigadas, Alaka Deshpande, Hervé Fleury

**Affiliations:** 1 CNRS, UMR 5234, Bordeaux, France; 2 Laboratoire de Virologie (WHO accredited), CHU de Bordeaux, Bordeaux, France; 3 JJ Hospital, Byculla, Mumbai, India; Centro de Biología Molecular Severo Ochoa (CSIC-UAM), Spain

## Abstract

**Background:**

We and others have shown that subtype C HIV-1 isolates from patients failing on a regimen containing stavudine (d4T) or zidovudine (AZT) exhibit thymidine-associated mutations (TAMs) and K65R which can impair the efficacy of Tenofovir (TDF) at second line. Depending on the various studies, the prevalence of K65R substitution as determined by the Sanger method ranges from 4 to 30%. Our aim was to determine whether ultra-deep pyrosequencing (UDPS) could provide more information than the Sanger method about selection of K65R in this population of patients.

**Methods:**

27 subtype C HIV-1 isolates from treated patients failing on a regimen with d4T or AZT plus lamivudine (3TC) plus nevirapine (NVP) or efavirenz (EFV) and who had been sequenced by Sanger were investigated by UDPS at codon 65 of the reverse transcriptase (RT). 18 isolates from naïve patients and dilutions of a control K65R plasmid were analysed by Sanger plus UDPS.

**Results:**

Analysis of Sanger sequences of subtype C HIV-1 isolates from naïve patients exhibited expected polymorphic substitutions compared to subtype B but no drug resistance mutations (DRMs). Quantitation of K65R variants by UDPS ranged from <0.4% to 3.08%. Sanger sequences of viral isolates from patients at failure of d4T or AZT plus 3TC plus NVP or EFV showed numerous DRMs to nucleoside reverse transcriptase inhibitors (NRTIs) including M184V, thymidine-associated mutations (TAMs) plus DRMs to non- nucleoside reverse transcriptase inhibitors (NNRTIs). Two K65R were observed by Sanger in this series of 27 samples with UDPS percentages of 27 and 87%. Other samples without K65R by Sanger exhibited quantities of K65R variants ranging from <0.4% to 0.80%, which were below the values observed in isolates from naïve patients.

**Conclusions:**

While Sanger sequencing of subtype C isolates from treated patients at failure of d4T or AZT plus 3TC plus NVP or EFV exhibited numerous mutations including TAMs and 8% K65R, UDPS quantitation of K65R variants in the same series did not provide any more information than Sanger.

## Introduction

We and others [1]–[2] have shown that subtype C HIV-1 isolates from Indian patients who fail on first-line HAART composed of stavudine (d4T) or zidovudine (AZT) plus lamivudine (3TC) plus nevirapine (NVP) or efavirenz (EFV) and according to WHO clinical and/or immunological criteria exhibit numerous drug resistance mutations (DRMs) to nucleoside reverse transcriptase inhibitors (NRTIs) and to non-nucleoside reverse transcriptase inhibitors (NNRTIs) in the reverse transcriptase (RT) part of the viral genome, including thymidine-associated mutations (TAMs) and K65R (the prevalence of which was around 8% in our series). When the RT sequences were introduced into the ANRS and Stanford algorithms, both algorithms showed that the DRMs of the first line induce a decreased susceptibility to tenofovir (TDF), an NRTI drug that is still used as second line in some southern countries. This has implications for public health because patients who fail with a first-line regimen including d4T or AZT plus 3TC plus NVP or EFV and who switch to 3TC plus TDF plus ritonavir-boosted lopinavir (LPV/RTV) will in fact not be fully susceptible to TDF and therefore to the second-line regimen. TAMs and K65R are known to induce partial or full resistance to TDF [3]. Regarding K65R, similar studies carried out in the same context of failure on a first-line regimen including d4T, AZT or dideoxyinosine (ddI) showed a prevalence of 4% in a South African population where subtype C was predominant [4], 10.9% in CRF02_AG plus G viruses in Nigeria [5], 14% in subtype C isolates from South Africa [6], 24% in Malawi with subtype C viruses [7] and up to 30% in Botswana [8].

From a molecular point of view, it has been demonstrated that the RT KKK nucleotide motif at codons 64, 65, 66 in reverse transcriptase of subtype C HIV-1 appears to lead to template pausing that facilitates the selection of K65R, even in isolates from untreated patients [9]–[12]. Moreover, it has also been shown that the KKK motif in this subtype can lead to PCR-induced K65R [13].

The aim of the present study was to clarify the prevalence of K65R in these subtype C isolates from patients failing on a first line including d4T or AZT. Since we had the prevalence of K65R by the Sanger sequencing method, we investigated K65R variants by ultradeep pyrosequencing (UDPS) in the same samples as those already used for bulk sequencing and sought whether UDPS provided additional knowledge to the Sanger results.

## Methods

Subtype C HIV-1 isolates from Indian patients failing (according to WHO clinical and/or immunological criteria) on a first-line treatment including d4T or AZT plus 3TC plus NVP or EFV were sequenced on RT by the Sanger method, and the sequences were recorded in the Los Alamos database (GenBank JF895621–JF895673). Among these samples, 27 were randomly selected for investigation by UDPS using the Roche GS Junior equipment. RNA extracted previously from the samples was used to amplify a short region of RT with primers including specific sequences for the GS Junior system (Table 1). The reverse transcription used SuperScriptIII RTPCR enzyme (Invitrogen, Carlsbad, CA) with 10 ul RNA, one cDNA synthesis cycle at 50°C for 30 min and 40 cycles of PCR amplification. The nested PCR used FastStart HiFi (Roche) with 2 ul of RTPCR product, 40 cycles of PCR amplification. Amplicons were purified by AMPure kit (Agencourt Biosciences), quantified using Quant-iT Picogreen (Invitrogen) and pooled at equimolar concentrations. Clonal amplification on beads (EmPCR) was performed using the 454 Life Science reagents that enable bidirectional sequencing, composed of a 30 cycle PCR amplification. DNA containing beads were recovered and UDPS was performed on the GS Junior sequencer (454 Life Sciences). Most of the samples had HIV viral loads >100,000 copies/mL (mean: 379,753 copies/mL; IQR: 11209–5,817,977 copies/mL) and at least 1,000 clonal sequencing reads were used for the analysis, allowing a 0.4% accuracy in the quantitation [14]. Positions studied were codon 65 and codon 70, which was chosen as a TAM position in NRTI-treated patients leading to K70R, because it has been frequently observed in the studied isolates and because 70R is considered to be a DRM and not at all a substitution potentially related to polymorphism.

**Table 1:** Primers used for GS Junior ultradeep sequencing of RT.

	sequence 5′-3′	HXB2 position
RT PCR GS Junior		
primer 5′	AGTAGGACCTACACCTGTCA	2480 to 2499
primer 3′	CTGTTAGTGCTTTGGTTCCTCT	3399 to 3420
Nested GS Junior		
primer 5′	GGCCATTGACAGAAGAAAAAATAAAAGC	2620 to 2647
primer 3′	GGGATGTGGTATTCCTAATTGAACTTCC	2813 to 2840

Since the viral isolates obtained at initiation of first-line therapy were not available, the data obtained by Sanger and UDPS in isolates from patients at failure were compared to those of subtype C isolates from 18 naïve patients. Some of their bulk sequences have been previously published by our group [15]. All codons were analysed by Sanger while potential polymorphism at codons 65 and 70 was investigated by both Sanger and UDPS. As a control, we used two subtype C MJ4 plasmids, one wild type and one bearing K65R (both provided by Mark Wainberg's group in Montreal). The UDPS results of the study are available in GenBank under accession number SRA 050640.

## Results

Table 2 shows the Sanger results of viral isolates from naïve patients and UDPS results of codons 65 plus 70 in these isolates, together with UDPS results of control plasmids for codons 65 and 70. Analysis of Sanger results for naïve patients showed an extensive polymorphism compared to subtype B without involvement of substitutions 65R and 70R. K70R was <0.4% by UDPS in all isolates from naïve patients. K65R ranged from <0.4% to 3.08% (mean 0.66±0.76 standard deviation, SD).

**Table 2:** RT polymorphic substitutions (Sanger) compared to reference HIV-1 B in isolates from naïve patients and amounts of K65R and K70R mutations in the same isolates plus control plasmids.

naive patients	RT sequence by Sanger method	K65R	K70R
1031	35Q, 39D, 48T, 48S, 60I, 73K, 73I, 122K, 135R, 162N, 173A, 174K, 177E, 178L, 200A, 207E, 211K, 214F, 245Q	<0.4%	<0.4%
1050	21I, 35T, 39E, 43K, 43R, 48T, 60I, 90I, 90V, 106M, 121Y, 135T, 135I, 158A, 158S, 162A, 162G, 170P, 170L, 173A, 177E, 179D, 200A, 207E, 211K, 214F, 221H, 221Y, 245Q	0,44%	<0.4%
1059	13R, 16N, 21G, 35T, 39D, 43R, 48T, 60I, 82R, 102Q, 107T, 107I, 121Y, 142I, 142V, 162A, 173A, 177E, 177D, 179I, 179V, 196E, 196G, 200A, 202V, 207G, 211K, 214F, 245Q, 250E	0,56%	<0.4%
1071	13K, 13R, 35T, 36A, 39E, 48T, 60I, 77I, 77F, 123E, 142V, 166R, 173A, 177E, 195L, 200A, 202V, 207A, 211K, 214F, 245Q	0,42%	<0.4%
1102	35T, 39N, 48T, 60I, 121H, 121Y,135R, 162A, 173A, 174K, 177E,200A, 207E, 211K, 214F, 245Q	0,71%	<0.4%
1113	20R, 35T, 36E, 36A, 39D, 60I,103K, 103N, 106M, 106V, 118I,121D, 121Y, 135T, 139S, 162A,165V, 173T, 177E, 179D, 179V, 200A, 207E, 211K, 245Q	1,22%	<0.4%
1114	28K, 32E, 35T, 36A, 39D, 48T, 60I, 121H, 173A, 174Q, 174R, 177E, 178L, 190R, 194H, 200A, 207E, 214C, 225L	<0.4%	<0.4%
1116	20R, 35T, 39E, 48T, 60I, 102K, 102Q, 121Y, 135T, 138A, 162C, 173A, 200A, 207E, 207A, 211K, 214L, 214F, 245Q	1,18%	<0.4%
1117	35T, 36E, 36A, 39D, 48T, 60I, 64R, 121Y, 121C, 166R, 173T, 173A, 175N, 175H, 177E, 200A, 207K, 214F, 245Q, 250E	0,35%	<0.4%
1121	35T, 39E, 48T, 60I, 110H, 110D,121Y, 135R, 173T, 177E, 200A,207E, 214F, 217P, 217S, 245Q,248R, 249R, 251V, 252G, 254F, 255K	1,33%	<0.4%
1123	10I, 35T, 36A, 39D, 48T, 60I, 121H, 139T, 139S, 173S, 174K, 177E, 178I, 178V, 200A, 207A, 211K, 214F, 245Q, 252C	<0.4%	<0.4%
1125	13N, 35T, 39D, 43R, 48T, 60I, 121H, 121Y, 135T, 162A, 173A, 174R, 177E, 200A, 207E, 211K, 214F, 245Q	0,85%	<0.4%
1129	35T, 36A, 39D, 48T, 60I, 122K,139A, 173T, 177E, 178M, 200A, 207E, 211K, 214F, 245Q	0,75%	<0.4%
1131	36A, 39E, 48T, 55T, 55P, 73K,73I, 123S, 138E, 138V, 173A,177E, 200A, 207E, 211K, 214F, 245Q	3,08%	<0.4%
1132	35T, 36A, 39D, 48T, 60I, 122P,162C, 166R, 173T, 173A, 177E,200A, 202I, 202V, 207E, 211K, 214F, 245Q	1,02%	<0.4%
1133	35T, 36A, 39D, 48T, 60I, 122P,123E, 161Q, 161L, 166R, 173A,177E, 200A, 207E, 211K, 214F, 220K, 220I, 245E, 248K	<0.4%	<0.4%
1135	35T, 36A, 39D, 48T, 49K, 49R,60I, 121C, 159I, 159V, 160C,160F, 162S, 162C, 165I, 173T,173A, 177E, 178M, 178L, 200A, 207E, 214F, 245Q	<0.4%	<0.4%
1139	35T, 39N, 48T, 60I, 102R, 104R, 121Y, 162A, 173A, 200A, 207K, 211K, 214F, 245Q	<0.4%	<0.4%
plasmid K65R 10%		94%	<0.4%
plasmid K65R 5%		2,30%	<0.4%
plasmid K65R 1%		0,90%	<0.4%

**Footnote to** **Table 2**. Two subtype C MJ4 plasmids, one with K65K (wild type) and one with K65R were used to amplify RT region before sequencing by UDPS. Amplicon pools were not prepared in equimolar concentrations but with different percentages of K65R mutation. The theoretical and observed values of K65R plasmid dilutions were 100%∶94%, 5%∶2.30% and 1%∶0.90%.

Regarding the 27 isolates from treated patients at failure, the drug resistance mutations (DRMs) according to the French ANRS algorithm and following bulk DNA sequencing (Sanger) are listed in Table 3. Most of them exhibited the M184V mutations to 3TC of the regimen plus TAMs to d4T and/or AZT and DRMs to NNRTIs. Only two samples (455 and 493) bore a K65R mutation, one (455) cumulating K65R and the Q151M nucleoside analog mutation (NAM). Table 4 compares the Sanger and UDPS data of these isolates at codons 65 and 70. Quantitation of K65R by UDPS ranged from <0.4% to 87%. In the two isolates with K65R by Sanger, percentages of K65R by UDPS were 27% and 87%. If we only consider positions 65 found not to have K65R with Sanger (25 samples), the quantities of K65R ranged from <0.4% to 0.80% (0.162±0.22 SD). K70R with UDPS ranged from <0.4% to 100%. Eight samples exhibited K70R with Sanger and the corresponding UDPS values ranged from 34.50 to 100%. Regarding positions 70 found not to have the K70R mutation with Sanger (19), all of them were <0.4% for 70R by UDPS except two samples (470 and 489 with values of 1.80 and 4.40% respectively).

**Table 3:** RT bulk sequences of 27 subtype C HIV-1 isolates failing on first line.

patient	RT sequence by Sanger method	patient	RT sequence by Sanger method
454	115F, 151M, 184V, 219Q, 90I, 181C, 221Y	479	41L, 44D, 67N, 75M, 184V, 215Y, 98G, 101E, 179I, 181C, 190A, 221Y
455	41L, 65R, 151M, 184V, 181V, 190A	480	41L, 67N, 69D, 70R, 184V, 210W, 215Y, 98G, 106M, 179I, 181C, 190A
456	67N, 70R, 184V, 215F, 219E, 98S, 181C	481	41L, 67N, 69insert, 75M, 184I, 210W, 215Y, 90I, 103N
461	41L, 44D, 69D, 184V, 90I, 179I, 181C	482	41L, 67N, 69D, 184V, 215Y, 101E, 179I, 188L, 190A
463	41L, 67N, 69D, 70R, 184V, 215Y, 188L, 221Y	485	no resistance mutation
464	184V, 98G, 101E, 181C, 190A	486	41L, 67N, 74V, 184V, 215Y, 101E, 138Q, 190S
465	41L,44D, 67N, 74V, 184V, 210W, 215Y, 101E, 179T, 181C, 190A	487	67N, 69D, 70R, 184V, 219Q, 98G, 179I, 190A
466	41L, 67N, 69D, 69N, 75M, 184V, 210W, 215Y, 101E, 179I, 190A	488	41L, 184V, 215Y, 103N, 225H
469	41L, 67N, 69N, 70R, 184V, 215F, 219E, 103N, 190A	489	74V, 184V, 215Y, 101E, 179I, 190C
470	no resistance mutation	493	65R, 75A, 219E, 179T, 181C, 190A, 221Y
471	41L, 184V, 215Y, 98G, 101E, 190A	495	41L, 184V, 210W, 215F, 90I, 103N
472	41L, 67N, 70R, 184V, 215Y, 219E, 181C	496	41L, 67N, 70R, 75M, 184V, 215F, 219Q, 106M, 190A
473	41L, 44D, 67N, 70R, 75M, 184V, 215Y, 103N, 190A	501	41L, 67N, 75M, 184V, 210W, 215Y, 101E, 190S
475	41L, 67N, 69D, 75M, 184V, 215Y, 101E, 179I, 188L, 190A		

DRMs are noted according to ANRS.

**Table 4:** Comparison of Sanger and UDPS sequencing results for RT codons 65 and 70; same isolates as in Table 3.

	K65R		K70R	
patient	Sanger	UDPS	Sanger	UDPS
454	N	<0.4%	N	<0.4%
455	O	87%	N	<0.4%
456	N	<0.4%	O	100%
461	N	<0.4%	N	<0.4%
463	N	<0.4%	O	34.6%
464	N	0.14%	N	<0.4%
465	N	<0.4%	N	<0.4%
466	N	0.5%	N	<0.4%
469	N	<0.4%	O	94%
470	N	<0.4%	N	1.8%
471	N	<0.4%	N	<0.4%
472	N	0.46%	O	81.6%
473	N	<0.4%	O	97.1%
475	N	<0.4%	N	<0.4%
479	N	0.24%	N	<0.4%
480	N	0.25%	O	84.4%
481	N	0.21%	N	<0.4%
482	N	<0.4%	N	<0.4%
485	N	<0.4%	N	<0.4%
486	N	0.3%	N	<0.4%
487	N	0.25%	O	90.1%
488	N	0.5%	N	<0.4%
489	N	<0.4%	N	4.4%
493	O	27%	N	<0.4%
495	N	0.4%	N	<0.4%
496	N	0.8%	O	98.15%
501	N	<0.4%	N	<0.4%

Sanger Y: presence of mutation; N: absence of mutation. UDPS: Frequency of mutations observed in samples.

## Discussion

### Sanger and UDPS results in isolates from naïve patients

Although UDPS has limitations particularly with regard to polymerization and pyrosequencing errors [13], [16], recent studies with different methods (UDPS, allele specific PCR) have shown that K65R is identified more frequently in subtype C HIV-1 from naïve patients [14], [17]. In our naïve patients, there was a clear difference between K70R (mean 0%) and K65R (mean 0.66%) (Table 2). Our data on position 65 are in agreement with those of Kozal et al [14], ranging from <0.4% to 1.33% apart from one sample (1131) at 3.08%. We were not expecting selection of K65R in this population of patients who were quite distant from primary infection and therefore from potential transmission of K65R mutants. For us, this naïve population was the basis of an evaluation of natural variation at codon 65 in subtype C HIV-1.

### Sanger and UDPS results of isolates from treated patients at failure

The Sanger results of isolates from treated patients were as expected with a predominance of M184V, numerous TAMs of pathway1 (M41L, D67N, K70R, L210W, T215Y/F) and DRMs to NNRTIs (mainly K101E, K103N, V106M, Y181C, G190A). As mentioned above, 2 isolates of the series exhibited a K65R substitution.

With regard to UDPS results at codon 70, the quantitative data were different from those recorded in naïve patients: 8 isolates exhibiting K70R with Sanger had UDPS K70R values above 34.60%, while 2 samples without K70R with Sanger (470 and 489) had K70R variants at quantities above the <0.4% background observed in naïve patients. We hypothesize that these isolates are undergoing a process of selecting K70R mutations. Regarding the K65R values apart the two samples with K65R by Sanger, the UDPS quantities of K65R variants were low and below those of isolates from naïve patients. Our results are not in accordance with those obtained by another group [18] using an allele- specific PCR which exhibited minority variants of K65R in four subtype C HIV-1 isolates out of 30 patients having received NRTIs at first line; it must be pointed out that this technique uses an intercalating dye and high-melt resolution assay which can be difficult to interpret due to genomic variability in the flanking region of codon 65.

K65R substitutions are generated in subtype C isolates from naïve patients due to the 64–65–66 motif. There are some constraints in experienced patients failing on a suboptimal regimen with d4T or AZT plus 3TC plus NVP or EFV. We first hypothesize that 184V, which was the most prevalent mutation observed in our series of treated patients at failure, has dampened the emergence of 65R as noted by others [3]. Second, there is an antagonism between TAMs and K65R, while the latter can be found in association with NAMs (Q151M) and is considered to be increasingly selected in the presence of DRMs to NNRTIs and particularly Y181C and G190A. As noted above, the prevalence of K65R in this clinical context of failure ranges from 4 to 30%. In our series, we estimate this prevalence to be 8% and the UDPS data did not reveal any process of K65R selection that cannot be assessed by Sanger sequencing.
